# Aesthetic Rehabilitation of Patients with Central and Peripheral Facial Palsy with Injectables (BNT-A, HA-Fillers and CaHa)

**DOI:** 10.3390/jcm15010388

**Published:** 2026-01-05

**Authors:** Athanasios Tsivgoulis, Eleftherios Stefas, Georgios Galatas, Georgia Papagiannopoulou, Stella Fanouraki, Maria-Ioanna Stefanou, Pinelopi Vlotinou, Christina Zompola, Georgios Tsivgoulis, Aikaterini Theodorou

**Affiliations:** 1 Attica Rehabilitation Center, 19018 Athens, Greece; nasost02@yahoo.gr; 2 Second Department of Neurology, National and Kapodistrian University of Athens, School of Medicine, “Attikon” University Hospital, 12462 Athens, Greece; georgiapap22@hotmail.com (G.P.); stelfanou@gmail.com (S.F.); marianna421@hotmail.co.uk (M.-I.S.); pakivlot@yahoo.com (P.V.); chriszompola@yahoo.gr (C.Z.); tsivgoulisgiorg@yahoo.gr (G.T.); 3 Department of Neurology, Democritus University of Thrace, 68100 Alexandroupolis, Greece; stefaspmr@gmail.com; 4 First Department of Internal Medicine, Sismanoglio General Hospital, 15126 Athens, Greece; giorgosgalatas@gmail.com; 5 Department of Occupational Therapy, School of Health and Welfare Sciences, University of West Attica, 12243 Athens, Greece

**Keywords:** facial palsy, rehabilitation, botulinum toxin, hyaluronic acid and calcium hydroxylapatite, injections

## Abstract

**Background:** Facial palsy constitutes a profoundly disabling condition, often leading to marked functional deficits and a decline in facial appearance, which substantially reduces the patient’s quality of life. A combined therapy of botulinum toxin (BoNTA), hyaluronic acid (HA) and calcium hydroxylapatite (CaHA) appears promising in the pharmacological approach of these patients. **Methods:** We reported our single center experience of patients with facial palsy, either of central or peripheral etiology who were treated with the combination of BoNTA, HA and CaHA, during a 6-month period (January 2025–June 2025). **Results:** Eight consecutive adult patients [mean age: 49.50 ± 7.95 years, 6 (75%) female] with facial palsy, either of central (4 patients) or peripheral (4 patients) etiology, received the combination of BoNTA, HA and CaHA. No serious adverse reactions were documented. Localized bruising and swelling at injection sites resolved without requiring any additional intervention. Facial Disability Index (FDI) was assessed both prior to and following treatment. The functional subscale increased from 65.63 ± 16.13 to 80.63 ± 10.50 (improvement rate = 24.4%, *p*-value = 0.002), while the psychosocial subscale increased from 63.00 ± 17.34 to 74.50 ± 10.89 (improvement rate = 18.3%, *p*-value = 0.004). Consequently, the total FDI score improved from 128.63 ± 28.92 to 155.13 ± 17.96 (overall improvement = 20.6%, *p*-value = 0.001). **Conclusions:** The present case series underscores the potential therapeutic role of CaHA as an adjunct to BoNTA and HA injections in patients with central or peripheral facial palsy.

## 1. Introduction

Facial palsy can result from either central (upper motor neuron) or peripheral (lower motor neuron) lesions [1]. Central facial palsy, most commonly caused by a contralateral stroke affecting the corticobulbar tract, produces weakness of the lower face while forehead movement and eye closure are preserved due to bilateral cortical innervation [2]. Clinically, this manifests as mouth asymmetry, nasolabial fold flattening, and impaired smiling or speech, with prognosis depending on lesion size, location, and rehabilitation [3]. In contrast, peripheral facial palsy, such as Bell’s palsy, involves the facial nerve distal to its nucleus, causing complete ipsilateral facial paralysis, including the upper face, and may affect eye closure, nasolabial fold, mouth movement, lacrimation, salivation, and taste [4]. Preservation of forehead movement remains a key feature distinguishing central from peripheral lesions.

Facial palsy constitutes a profoundly disabling condition, often leading to marked deficits in facial expressivity and functional disturbances of speech and mastication. Chronic manifestations may include persistent facial asymmetry, aberrant reinnervation with synkinesis, and hyperactivity of the unaffected hemiface [5]. These factors can collectively diminish the overall facial aesthetics [6]. The combination of functional deficits and a decline in facial appearance substantially reduces the patient’s quality of life (QoL), often resulting in social withdrawal, loss of self-esteem, and adverse psychological outcomes [7,8].

Prolonged and frequent treatment sessions are often necessary. During the acute and subacute phases, a comprehensive rehabilitation program is recommended, incorporating physiotherapy, speech therapy, and adjunctive therapeutic modalities [9,10]. In the chronic phase, a combined therapeutic approach involving pharmacological intervention (botulinum toxin) and dermal filler application represents the optimal strategy, as it addresses both the static and dynamic components of facial expression [11].

Botulinum toxin A (BoNTA) injection has emerged as a foundational therapy for facial synkinesis. Numerous studies have demonstrated its effectiveness, improving symmetry, reducing involuntary contractions, and enhancing facial animation [12,13]. BoNTA has been used both as a standalone therapy and in combination with soft-tissue fillers to address residual asymmetries that persist after neuromuscular rebalancing [14]. Hyaluronic acid (HA) fillers are the most commonly reported adjunctive agents. Their reversible volumizing properties allow for the correction of contour irregularities, compensation for soft-tissue atrophy, and restoration of structural support for periorbital and perioral regions frequently affected by synkinetic movement patterns [15,16]. Combining HA fillers with BoNTA may produce superior aesthetic and functional outcomes compared with either modality alone, particularly in patients with volume loss or ligamentous imbalance [17].

Recently, calcium hydroxylapatite (CaHA) has been also proposed as adjunctive option in both reconstructive and aesthetic literature [18,19]. This therapeutic approach is supported by its principal mechanisms of action, including immediate lifting capacity and delayed biostimulatory effects through fibroblast activation and collagen neogenesis. These characteristics may enhance skin quality, ligamentous tension, and soft-tissue architecture [20]. The effect duration has been estimated at 12 to 24 months.

Collectively, these injectable therapies represent a multimodal approach targeting distinct pathophysiological components of facial synkinesis. When integrated appropriately, they may improve resting symmetry, reduce involuntary contractions, and optimize dynamic facial expressions. However, standardized protocols remain understudied, and the existing literature is heterogeneous in terms of injection techniques, dosing strategies, and outcome measures.

We sought to evaluate the effect of combining CaHA with BoNTA and HA dermal fillers on aesthetic improvement as well as the functional and psychological outcomes in consecutive patients with facial palsy of either central or peripheral etiology.

## 2. Materials and Methods

The datasets generated and/or analyzed during the current study are available from the corresponding author upon reasonable request.

### 2.1. Participants

Consecutive adult (older than 18 years) patients with facial palsy, either of central or peripheral etiology, were recruited prospectively between January 2025 and June 2025 at the Second Department of Neurology of National & Kapodistrian University of Athens at “ATTIKON” University Hospital in Athens. The patients presented either at our stroke units or at our outpatient stroke or general neurology clinics and they underwent a combined treatment with BoNTA, HA, and CaHA. Detailed medical and patient history intake and thorough clinical examination were performed in all patients of this registry to identify the underlying etiology and relevant clinical signs. Moreover, patients who did not provide consent to participate in the current study were excluded.

### 2.2. Treatment Strategy and Follow-Up Evidence

The treatment protocol is segmented into three stages. During the initial stage (Supplementary Table S1), the patient undergoes a comprehensive clinical assessment, evaluating both static and dynamic facial features, while the patient performs functional movements such as smiling, speaking, whistling, chewing, and drinking. The severity of facial palsy is assessed based on the House–Brackmann Garding System [21]. This is followed by an exploration of the functional and daily impact of the condition, focusing on difficulties with eating, speech, facial expression, and the social or psychological challenges the patient may experience. A thorough medical background, including any previous injectable treatments and relevant medical history or comorbidities, is reviewed. Psychosocial evaluation is equally important, assessing the emotional and professional burden of the condition and ensuring that the patient’s motivations and expectations regarding functional and aesthetic improvement are realistic. Clear communication of the proposed procedures, techniques, safety considerations, follow-up needs, and expected outcomes is essential, along with obtaining informed consent and authorization for photo or video documentation.

Once written informed consent is obtained, the patient proceeds to the next stage. Based on the primary muscles involved, a clinical objective is defined, and a predetermined approach can be followed (more details are provided in Supplementary Table S2). Management of facial paralysis involves a targeted, region-by-region approach that considers the main muscles involved and the functional imbalance between the healthy and paralyzed sides. In the frontal and periorbital areas, the focus is on harmonizing brow position, improving eyelid closure, and reducing asymmetric muscle activity. Midface treatment aims to rebalance the smile by supporting weakened tissues and controlling hyperactivity on the opposite side. The perioral region is addressed to restore lip competence and improve commissure symmetry, while work on the chin and lower face helps counteract dominant depressor pull and enhance contour. When needed, the neck is also treated to soften platysmal asymmetry. Together, these coordinated measures help restore facial balance and function.

During the second phase, the patient undergoes treatment with a combination of BoNTA, HA, and CaHA. Dosage determination is tailored to the patient’s individual needs. According to the treatment plan and the clinical response of the patient, up to three or four follow-up sessions combined with re-administration of the toxin, HA, and/or CaHA injection can be performed. During the final stage, a comprehensive clinical evaluation is performed.

Moreover, assessment using the Facial Disability Index (FDI) is performed at baseline assessment and following treatment completion, with all findings systematically documented (Supplemental Figure S1) [22,23]. In addition to the total FDI score, the functional and psychosocial subscales are evaluated. The functional subscale assesses the physical limitations caused by facial nerve dysfunction, including impairment in mastication and oral competence, articulation, ocular protection, and voluntary facial motor function. The psychosocial subscale measures the emotional and social effects of facial impairment, such as changes in self-confidence, social interaction, and overall well-being.

### 2.3. Ethical Approval and Patient Consent

This study was conducted in accordance with the Declaration of Helsinki principles and was approved by the Ethics Committee of “ATTIKON” University Hospital (Decision Number: EBΔ 354/15-4-2024). Written informed consent for publication of this study was obtained from all patients in accordance with the current version of the Declaration of Helsinki.

### 2.4. Statistical Analysis

We assessed the pooled prevalence of baseline characteristics of all patients included in the present case series. Continuous variables were summarized using the mean and corresponding standard deviation (SD) when a normal distribution was confirmed, with normality assessed using the Shapiro–Wilk test. In cases where continuous variables demonstrated a skewed or non-normal distribution, data were reported as the median accompanied by interquartile ranges (IQRs). Categorical variables were presented as the number of patients with the corresponding percentages. Comparisons between different variables were performed using the paired t-test, as appropriate for within-subject analyses. Statistical significance was defined a priori as a two-sided *p*-value < 0.05. All statistical analyses were carried out using the R statistical software environment (version 2025.05.0 + 496) [24].

## 3. Results

**Figure 1:**
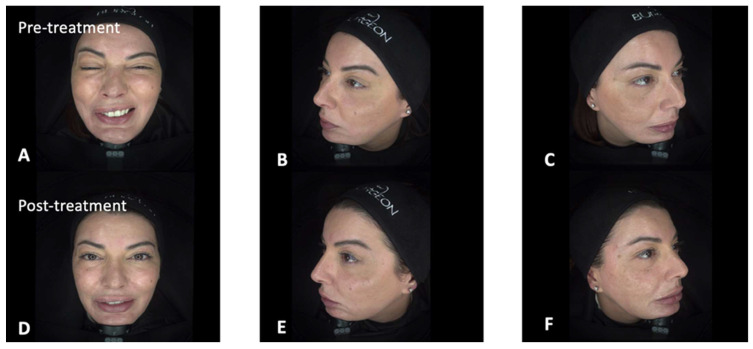
Representative patient case with peripheral facial palsy. Figure Legend: 42-year-old female patient with a peripheral facial nerve palsy following right-ear cyst removal performed in childhood (iatrogenic). Pre-treatment images capturing the profile and bilateral facial sides (Panels **A**–**C**), with the characteristic right facial paralysis, major facial asymmetry and right brow ptosis. Marked clinical improvement was noticed, following combined treatment with BoNTA, HA, and CaHA (Panels **D**–**F**).

**Figure 2:**
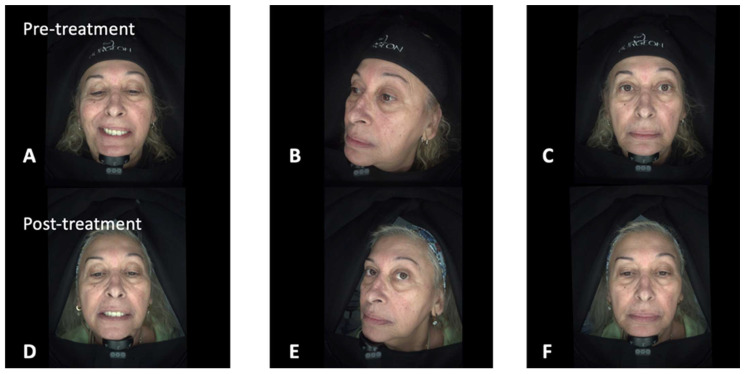
Representative patient case with central facial palsy. Figure Legend: 63-year-old patient with left facial hemiparesis, facial asymmetry, dysarthria, and slight food and liquid leakage following an ischemic stroke in the distribution of the right middle cerebral artery. Pre-treatment images capturing the profile and the left facial side (Panels **A**–**C**). Marked clinical improvement was noticed, following combined treatment with BoNTA, HA, and CaHA (Panels **D**–**F**).

Between January 2025 and June 2025, a total of eight patients [6 (75%) females, mean age (SD): 49.50 (7.95)] with either peripheral (4 patients) or central (4 patients) facial palsy were treated with BoNTA, HA, and CaHA (Table 1). Facial paralysis severity was predominantly high, with 50% (*n* = 4) of patients presenting with Grade V (severe dysfunction). Grade IV and Grade III dysfunction were observed in 25% (*n* = 2) and 12.5% (*n* = 1) of cases, respectively, while 12.5% (*n* = 1) of patients had Grade VI (total paralysis). No patients presented with Grade I or Grade II dysfunction. In summary, a mean of 43 (±22) UI of BoNTA, 5.00 (±1.07) ml of HA and a mean of 1.63 (±0.52) boxes of CaHA (commercial product: Novuma^®^, no financial interest) were used for each patient. In the study cohort, the median time from facial palsy onset to initiation of treatment was 42 months (IQR, 13–106 months). The median interval between treatment sessions was 3.5 weeks (IQR, 3–4 weeks). Representative clinical cases of patients affected by either peripheral or central facial palsy are illustrated in Figure 1 and Figure 2. A comprehensive overview of the treatment sessions administered to each patient is provided in Supplementary Table S3. Patient 1 (Figure 1), diagnosed with peripheral facial nerve palsy, initially underwent neuromodulation aimed at regulating overactive muscle groups associated with facial synkinesis. This initial intervention was followed by a series of progressive filler and contouring sessions designed to improve facial symmetry, particularly involving the lips, midface, and jawline. Patient 2 (Figure 2), who presented with left-sided facial paralysis secondary to an ischemic stroke, followed a comparable treatment strategy. The therapeutic plan included an initial session focused on muscle modulation, a subsequent session combining touch-up neuromodulation with volumetric filler restoration on the paretic side, and a final session dedicated to refining perioral, zygomatic, and temple contours. In both cases, treatments were deliberately spaced across multiple sessions to allow for gradual restoration of facial balance, optimization of functional outcomes, and enhancement of overall aesthetic harmony. No serious adverse reactions were documented following the administration of this combined treatment. The only recorded adverse events were mild and transient, consisting of localized bruising and swelling at the injection sites. These minor reactions resolved spontaneously within a few days without the need for additional medical intervention.

**Figure 3:**
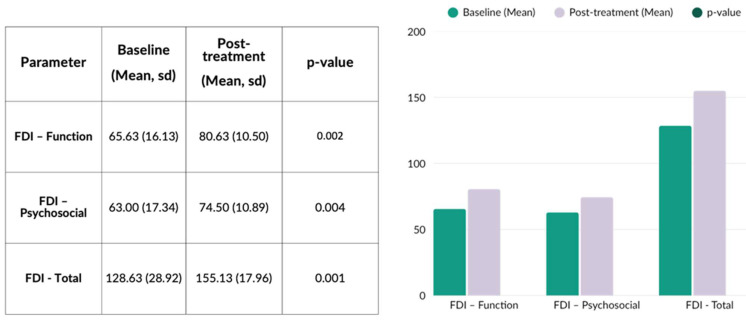
Facial Disability Index (FDI) assessment prior to and following treatment. Figure Legend: The synergistic use of BoNTA, HA, and CaHA yielded notable gains across the functional and psychosocial subscales, along with an overall increase in the FDI total score.

FDI was assessed both before treatment initiation and at the concluding clinical assessment. Overall, FDI showed significant improvement following treatment (Figure 3). More specifically, the functional subscale increased from 65.63 ± 16.13 to 80.63 ± 10.50 (improvement rate = 24.4%, *p*-value = 0.002), while the psychosocial subscale increased from 63.00 ± 17.34 to 74.50 ± 10.89 (improvement rate = 18.3%, *p*-value = 0.004). Consequently, the total FDI score improved from 128.63 ± 28.92 to 155.13 ± 17.96 (overall improvement = 20.6%, *p*-value = 0.001).

## 4. Discussion

In the present study, we investigated consecutive patients suffering from facial palsy of either central or peripheral etiology, who were treated with a combined therapeutic protocol consisting of BoNTA, HA, and CaHA injections. The results of our study showed that this multimodal therapeutic approach appears to be a safe and potentially beneficial intervention for addressing both the functional impairments and the psychosocial challenges linked to facial palsy. Specifically, treatment was associated with improvements in facial symmetry, reduction of facial synkinesis, restoration of soft-tissue volume, and enhancement of overall facial aesthetics. These physical improvements were accompanied by a positive impact on patient self-perception, confidence, and psychosocial well-being. However, the placebo effect and natural course of recovery cannot be definitely excluded.

To the best of our knowledge, this is the first study to specifically evaluate the adjunctive benefits of CaHA administered in addition to BoNTA and HA in patients affected by facial palsy. Previous studies have already shown the positive impact of combined therapy of BoNTA and HA on psychosocial and functional aspects, promoting facial symmetry [17]. Dermal fillers and BoNTA belong to minimally invasive procedures and they have been used alone or in combination, tailored to the individual needs of each patient [25]. In addition to addressing asymmetry, the combined use of BoNTA and dermal fillers has also been shown to exert beneficial effects in the management of facial synkinesis, contributing to improved muscular coordination and facial balance [26].

In addition, the nowadays available CaHA-based technology acts as a novel biostimulator that activates the skin’s intrinsic regenerative mechanisms by stimulating the endogenous production of key structural proteins, particularly collagen and elastin. Through this biostimulatory activity, CaHA promotes deep dermal remodeling and regeneration, resulting in sustained improvements in skin elasticity, firmness, and overall tissue quality [27]. CaHA is commercially available as an injectable dermal filler composed of biocompatible microspheres suspended within a gel carrier, which provides immediate volumizing effects following administration. The CaHA microspheres are evenly and smoothly distributed within the target tissue, where they act as a scaffold for cellular activity. Over the subsequent weeks, these microspheres stimulate local fibroblast activation and proliferation, thereby promoting collagen production through neocollagenesis [28]. This biologically driven regenerative process typically begins approximately four weeks post-injection and has been shown to persist for at least twelve months, contributing to long-lasting structural support and tissue quality improvement [28]. There are accruing data showing favorable durability and low complication rates associated with CaHA use for facial soft-tissue augmentation, including the treatment of wrinkles and folds, correction of lipoatrophy, and restoration of facial contour defects [28].

In accordance with the available literature, CaHA combined with BoNTA and HA appears to represent a safe and well-tolerated therapeutic option [29]. In our case-series, no serious adverse reactions, were observed following the administration of this combined treatment approach, including the absence of clinically significant complications such as infections, nodule formation, or granulomatous reactions. The absence of treatment-related adverse effects may be attributed not only to the favorable and well-established safety profiles of the agents employed but also to the careful patient selection and the administration of these injectable therapies by experienced and appropriately trained physicians.

Certain limitations of this study should be acknowledged. First, the small sample size of our cohort (n = 8) combined with a short, not-predefined follow-up time predisposes to selection bias and limits the feasibility of conducting in-depth statistical analyses to assess predictors of duration of the therapeutic effect and probable clinical deterioration. Second, the individualized treatment approach leading to treatment heterogeneity prevents drawing firm conclusions on required therapeutic doses for achieving successful therapeutic benefit and clinical outcome. Moreover, the lack of stratified analysis based on the type of facial paralysis (central versus peripheral) represents another limitation of the study. Although all patients received the same treatment protocol, the limited sample size within each subgroup precluded a meaningful comparison of outcomes according to paralysis type. Consequently, potential differences in treatment response related to the underlying etiology of facial paralysis could not be fully evaluated.

## 5. Conclusions

In conclusion, the results of the present case series highlight the potential therapeutic value of CaHA as an adjunctive therapy to BoNTA and HA injections among patients affected by either central or peripheral facial palsy. The addition of CaHA appears to contribute to both functional and aesthetic improvements, potentially enhancing facial symmetry, soft-tissue quality, and overall patient satisfaction. Nevertheless, these preliminary observations should be interpreted with caution, as they require independent validation through larger, multicenter, and preferably international prospective cohort studies with standardized outcome measures and sufficiently long follow-up periods to confirm efficacy, durability, and long-term safety.

## Figures and Tables

**Table 1:** Baseline Characteristics and treatment details.

Variable	Total (N = 8)
Sex, female (n, %)	6 (75%)
Age (years), mean (sd)	49.50 (7.95)
Diagnosis	
- Stroke (n, %) - Bell’s palsy (n, %)	4 (50%) 4 (50%)
Degree of facial paralysis	
- Grade I (Normal), (n, %) - Grade II (Mild Dysfunction), (n, %) - Grade III (Moderate Dysfunction), (n, %) - Grade IV (Moderately Severe), (n, %) - Grade V (Severe Dysfunction), (n, %) - Grade VI (Total Paralysis), (n, %)	0 (0%) 0 (0%) 1 (12.50%) 2 (25%) 4 (50%) 1 (12.50%)
Botulinum Toxin A (units), mean (sd)	43.12 (22.19)
Filler (Hyaluronic Acid) volume (mL), mean (sd)	5.00 (1.07)
Number of Novuma^®^ boxes (CaHA) used per patient, mean (sd)	1.63 (0.52)
Time interval between facial palsy onset and treatment initiation (Months), Median (IQR)	42 (13–106)
Time interval between treatment sessions (weeks), Median (IQR)	3.5 (3–4)

Abbreviations: CaHA: Calcium Hydroxylapatite, IQR: Interquartile Range, sd: standard deviation.

## Data Availability

All data needed to evaluate the conclusions in the paper are present in the main manuscript. Additional data related to this paper may be requested from the corresponding author, upon reasonable request.

## Supplementary Materials

The following supporting information can be downloaded at: https://www.mdpi.com/article/10.3390/jcm15010388/s1, Table S1: Baseline Evaluation and Administrative Procedures; Table S2: Therapeutic Plan Based on Muscle Involvement; Table S3: Overview of treatment sessions required for each patient; Figure S1: Facial Disability Index.
