# Supplementary material for: Aesthetic Rehabilitation of Patients with Central and Peripheral Facial Palsy with Injectables (BNT-A, HA-Fillers and CaHa)
Source: J Clin Med. 2026 Jan 5;15(1):388. doi: 10.3390/jcm15010388 (PMC12786528; doi:10.3390/jcm15010388)
Supplement: Supplementary file 1 [file jcm-15-00388-s001.zip › jcm-4052052-supplementary.pdf]

SUPPLEMENTAL MATERIAL

TITLE: Aesthetic Rehabilitation of patients with central and peripheral facial palsy with injectables (BNT-A, HA-fillers and Ca-Ha)

Supplementary Tables:

- **Supplementary Table-S1:** Baseline Evaluation and Administrative Procedures
- **Supplementary Table-S2:** Therapeutic Plan Based on Muscle Involvement
- **Supplementary Table-S3:** Overview of treatment sessions required for each patient.

Supplementary Figures:

- **Supplementary Figure-S1.:** Facial Disability Index

References used in the Supplement.

SUPPLEMENTAL TABLES

Supplementary Table-S1: Baseline Evaluation and Administrative Procedures

Category	Key Points
**1. Clinical Observation**	• Assess static & dynamic facial features.<br>• Observe patient while smiling, talking, whistling, masticating, drinking water.<br>• Identify type and degree of asymmetry.
**2. Functional & Daily Impact**	• Ask about daily functional challenges (eating, speaking, facial movement).<br>• Discuss social and psychological difficulties related to the condition.
**3. Medical Background**	• Document any previous injectable treatments.<br>• Review patient's medical history relevant to facial paralysis, as well as comorbidities.
**4. Psychosocial Evaluation**	• Assess emotional and professional burden.<br>• Confirm patient's motivation for appearance and function improvement.
**5. Patient Information & Consent**	• Explain procedure, follow-ups, techniques, safety, and realistic expectations.<br>• Obtain signed consent form and GDPR/photo-sharing consent.
**6. Clinical Documentation**	• Have patient fill out the Facial Disability Index (FDI).<br>• Capture standardized photos (static, dynamic, side).<br>• Record ~60 sec video where patient describes their condition and challenges.

Supplementary Table-S2: Therapeutic Plan Based on Muscle Involvement

Region	Main Muscles Involved	Clinical Goal	Technique / Approach
**Frontal Region**	Frontalis, Corrugator Supercilii, Procerus	Equalize brow elevation, reduce contralateral hyperactivity	1-5 units BoNT per injection point on healthy side; small CaHA/HA bolus (0.1–0.2 ml) on paralyzed side for volume support. Temple enhancement with CaHa/HA up to 1-2ml per side (cannula use is strongly advised)
**Periorbital**	Orbicularis Oculi	Improve eyelid closure, reduce spastic asymmetry, reduce twitching	5-10 unit BoNT lateral orbicularis on healthy side; microdroplet CaHA+HA (0.05–0.1 ml) around lateral canthus
**Midface (Zygomatic region)**	Zygomaticus Major/Minor, Levator Labii Superioris	Rebalance smile dynamics, stimulate collagen in atrophic tissues	0.4–0.8 ml diluted CaHA+HA on paralyzed side along zygomatic vector, consider BNT-A on healthy side when synkinesis is present
**Perioral**	Orbicularis Oris, Buccinator	Restore lip competence, symmetry of oral commissure, support dropping mouth corner	0.1–0.3 ml per injection point; BoNT 1–2 units to depressor on healthy side. Support of dropping mouth corner with 0,5ml HA, lip asymmetry correction with 0,5-1ml of HA
**Chin & Lower Face**	Depressor Anguli Oris, Mentalis, Platysma	Correct downward pull, reduce platysmal dominance	BoNT 10-15 units on healthy side; 0.2-0,8 ml CaHA+HA at mandibular border (biostimulation lines)
**Neck (Optional)**	Platysma	Soften neck asymmetry	Superficial diluted CaHA (hyperdilution 1:1–1:2 with saline + lidocaine) by cannula

Supplementary Table-S3: Overview of treatment sessions required for each patient.

Session	Timing	Botulinum Toxin (BNT-A)	Hyaluronic Acid (HA)	Novuma	Notes / Target Areas
**Patient #1: Peripheral facial nerve palsy**					
**1st Session**	Baseline	230 IU aboBNT-A	–	–	Frontalis, orbicularis, zygomaticus, platysma, perioral (left side) – considering synkinesis (facial twitching)
**2nd Session**	+3 weeks	No touch-up	3 ml (lips, marionette lines, nasolabial folds, temples)	1 box (bilateral jawline)	Aimed to enhance facial symmetry
**3rd Session**	+2 weeks	–	3 ml (right facial side and lips)	1 box (jawline contour and marionette area)	Final contour refinement
**Patient #2: Isch. Stroke left spastic hemiparesis left facial paralysis and asymmetry**					
**1st Session**	Baseline	20 IU onaBNT-A	–	–	Frontalis, orbicularis, zygomaticus, platysma, perioral (left side) – taking into consideration synkinesis (facial twitching)
**2nd Session**	+3 weeks	10 IU as touch-up	3 ml (lips, marionette lines, nasolabial folds, temples left)	1 box (zygoma and jawline left, marionette left)	To enhance facial symmetry, restore the paretic side
**3rd Session**	+3 weeks	–	3 ml (perioral and lips, zygoma both sides)	1 box (temple left and zygoma bilateral)	Final contour refinement

SUPPLEMENTAL FIGURES

Supplementary Figure-S1.: Facial Disability Index [1]

FACIAL DISABILITY INDEX (FDI)

Name: ______________________________ Date: ______________

Please choose the most appropriate response to the following questions related to problems associated with the function of your facial muscles.
For each question, consider your function **during the past month**.

Office Use Only
Score / Goal

Physical Function

1. How much difficulty did you have keeping food in your mouth, moving food around your mouth, or getting food stuck in your cheek?
   Usually did with:
   5 = No difficulty 2 = Much difficulty
   4 = A little difficulty 1 = Usually did not eat because of health
   3 = Some difficulty 0 = Usually did not eat because of other reasons

1. ________

2. How much difficulty did you have drinking from a cup?
   Usually did with:
   5 = No difficulty 2 = Much difficulty
   4 = A little difficulty 1 = Usually did not eat because of health
   3 = Some difficulty 0 = Usually did not eat because of other reasons

2. ________

3. How much difficulty did you have saying specific sounds while speaking?
   Usually did with:
   5 = No difficulty 2 = Much difficulty, slurring most of speech
   4 = A little difficulty 1 = Usually did not eat because of health
   3 = Some difficulty 0 = Usually did not eat because of other reasons

3. ________

4. How much difficulty did you have with your eye tearing excessively or becoming dry?
   Usually did with:
   5 = No difficulty 2 = Much difficulty
   4 = A little difficulty 1 = Usually did not eat because of health
   3 = Some difficulty 0 = Usually did not eat because of other reasons

4. ________

5. How much difficulty did you have with brushing your teeth or rinsing your mouth?
   Usually did with:
   5 = No difficulty 2 = Much difficulty
   4 = A little difficulty 1 = Usually did not eat because of health
   3 = Some difficulty 0 = Usually did not eat because of other reasons

5. ________

Total: ________

(___ - 5) / 5 x 25 = ________ Physical Score
(___ - 5) / 5 x 25 = ________ Physical Score Goal
For office use only

Please Turn Over for Part 2

Facial Disability Index – Part 2

Please choose the most appropriate response to the following questions related to problems associated with the function of your facial muscles.
For each question, consider your function **during the past month**.

Office Use Only
Score / Goal

Social / Well-being Function

6. How much time have you felt calm and peaceful?
   6 = All of the time 3 = Some of the time
   5 = Most of the time 2 = A little bit of the time
   4 = A good bit of the time 1 = None of the time

6. ________

7. How much of the time did you isolate yourself from people around you?
   1 = All of the time 4 = Some of the time
   2 = Most of the time 5 = A little bit of the time
   3 = A good bit of the time 6 = None of the time

7. ________

8. How much of the time did you get irritable toward those around you?
   1 = All of the time 4 = Some of the time
   2 = Most of the time 5 = A little bit of the time
   3 = A good bit of the time 6 = None of the time

8. ________

9. How often did you wake up early or wake up several times during your nighttime sleep?
   1 = Every night 4 = Some nights
   2 = Most nights 5 = A few nights
   3 = A good number of nights 6 = No nights

9. ________

10. How often has your facial function kept you from going out to eat, shop, or participate in family or social activities?
    1 = All of the time 4 = Some of the time
    2 = Most of the time 5 = A little bit of the time
    3 = A good bit of the time 6 = None of the time

10. ________

Total: ________

For office use only
(___ - 5) / 5 x 20 = ________ Social/Wellbeing Score
(___ - 5) / 5 x 20 = ________ Social/Wellbeing Score Goal

Physical (____) + Social (____) = (_______/ 200) total FDI Score

Physical (____) + Social (____) = (_______/ 200) total FDI Score Goal

References used in the Supplement.

1. Pavese C, Giordano A, Dalla Toffola E, et al. Facial Disability Index in Adults With Peripheral Facial Palsy: Rasch Analysis and Suggestions for Refinement. *Arch Phys Med Rehabil* 2022; 103: 1544-1550. 20211210. DOI: 10.1016/j.apmr.2021.10.030.
